# The cellular response to plasma membrane disruption for nanomaterial delivery

**DOI:** 10.1186/s40580-022-00298-7

**Published:** 2022-02-01

**Authors:** Gaëlle Houthaeve, Stefaan C. De Smedt, Kevin Braeckmans, Winnok H. De Vos

**Affiliations:** 1 Laboratory of Cell Biology and Histology, Department of Veterinary Sciences, University of Antwerp, Antwerp, Belgium; 2 Laboratory of General Biochemistry and Physical Pharmacy, Ghent University, Ghent, Belgium

**Keywords:** Intracellular delivery, Nanotechnology, Plasma membrane disruption, Cellular homeostasis

## Abstract

Delivery of nanomaterials into cells is of interest for fundamental cell biological research as well as for therapeutic and diagnostic purposes. One way of doing so is by physically disrupting the plasma membrane (PM). Several methods that exploit electrical, mechanical or optical cues have been conceived to temporarily disrupt the PM for intracellular delivery, with variable effects on cell viability. However, apart from acute cytotoxicity, subtler effects on cell physiology may occur as well. Their nature and timing vary with the severity of the insult and the efficiency of repair, but some may provoke permanent phenotypic alterations. With the growing palette of nanoscale delivery methods and applications, comes a need for an in-depth understanding of this cellular response. In this review, we summarize current knowledge about the chronology of cellular events that take place upon PM injury inflicted by different delivery methods. We also elaborate on their significance for cell homeostasis and cell fate. Based on the crucial nodes that govern cell fitness and functionality, we give directions for fine-tuning nano-delivery conditions.

## Introduction

Various therapeutic and diagnostic applications demand the introduction of specific nanomaterials into the cell’s interior. For instance, in cell-based therapies, the genetic engineering of cells requires the introduction of nucleic acids (e.g., mRNA) and/or proteins (e.g., Cas9 nuclease) into the cytoplasm or nucleus. Labelling of cells, whether it is for fundamental studies or for diagnostic applications, requires intracellular delivery of contrast agents. The plasma membrane (PM) is however impermeable to most of those functional molecules, demanding intracellular delivery technologies. Such methods are broadly classified into carrier-mediated or membrane disruption-mediated approaches. Carrier-mediated approaches rely on the packaging of cargo molecules within a carrier, which enters the cell via endocytosis or by fusion with the PM. The design of the carrier in terms of size, shape, hydrophobicity, surface charge and surface modifications determines where the cargo will be trafficked to inside the cell. Downsides of carrier-mediated approaches include restricted cargo-carrier combinations, carrier-induced toxicity and overall poor delivery efficiency. Membrane disruption-mediated approaches on the other hand, deliver cargo directly to the cytosol by creating transient pores in the PM. PM permeability is induced mostly by physical stimuli, such as electrical fields, mechanical forces, or light, which can be very well controlled. Intracellular delivery by membrane disruption has the advantage that is a quite universal approach that works independent of the type of cell or cargo molecules. A limitation, on the other hand, is that it is mostly limited to in vitro or ex vivo applications, such as genetic engineering of cell therapy products, while carrier-based delivery is more suited for in vivo therapies.

In the past decade, the field of membrane disruption methods for intracellular delivery has grown exponentially owing to advances in nanotechnology, including the emergence of various nanomaterials and nanofabrication methods [1]. One noteworthy example is the combination of nanosensitizers with laser irradiation, which has gained significant interest due to its ability to permeabilize the PM of both adherent and suspension cells with high throughput and spatiotemporal selectivity [2–6]. While most studies on intracellular delivery technologies focus on achieving high delivery efficiencies with minimal acute cytotoxicity, recent studies show that surviving cells can suffer from more subtle effects, such as alterations in morphology and functionality. For example, after permeabilization by a strong electrical field, sustained increases of cell activation markers [7] and loss of cell adhesion proteins [8] have been observed. Therefore, to expedite development of refined intracellular delivery methods based on PM disruption, a deeper understanding is required of how cells respond to and recover from such methods.

In this review, we will first describe the main PM disruption methods used for intracellular delivery. Next, we provide a short overview of the cellular response to PM damage. We then break down the observed cellular effects into the different time scales at which they operate, making a distinction between general effects and those that are specific to a particular type of PM disruption method. Finally, we discuss how these insights may contribute to improving physical delivery methods.

## Membrane disruption-mediated techniques for intracellular delivery

**Fig. 1:**
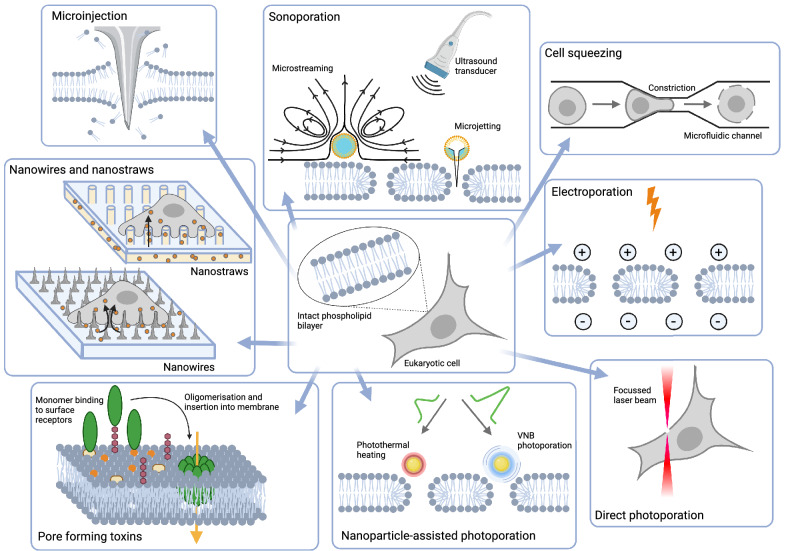
An overview of the main techniques for PM disruption. Explanation of the individual techniques can be found in the main text

Membrane disruption-mediated techniques perturb the integrity of the PM by generating lesions of different sizes and shapes. Except for pore-forming toxins, all are based on physical stimuli. Here below, we will give a concise overview of the main methods (Fig. 1) and in Table 1 we list their major properties. For a more comprehensive explanation of these techniques, the reader is referred to Stewart et al. (2018).

**Table 1:** Major properties of nanotechnologies for intracellular delivery

	Efficiency	Toxicity	Throughput	Precision at single cell level	Applicability	Pore size	Cargo size that can be delivered	Mechanisms of membrane permeabilization	Documented cellular responses
Nanowires and nanostraws	Low	Low	High	Medium	In vitro*, *ex vivo	≤ 100 nm	Several MDa	Combination of direct penetration and stimulated endocytosis	A, D, E
Pore forming toxins	High	High	High	Low	In vitro	15–30 nm	Up to 150 kDa	Membrane insertion	A, E
Electroporation	High	Medium	High	Low	In vitro*, *ex vivo	1–400 nm	Several MDa	Formation of electropores	B, C, F
Sonoporation	Medium	High	High	Low	In vitro*, *in vivo	50–250 nm	Several MDa	Different types of mechanical forces including shock waves and shear stress	A, B, C, E, F
Microfluidic cell squeezing	High	Low	High	Medium	In vitro*, *ex vivo	ND	15 nm AuNP, QD and antibodies	Mechanical deformation	ND
Direct laser-induced photoporation	High	Medium	Low	High	In vitro	80–160 nm	Several MDa	A combination of thermal, mechanical and chemical effects	A, B, C
Nanoparticle-mediated photoporation	High	Low	High	High	In vitro*, *ex vivo	10–500 nm	100–1000 s of kDa	Photothermal heating, high-pressure shockwaves or liquid jet formation	A, B, C, E
PEN photoporation	High	Low	High	High	In vitro*, *ex vivo	ND	up to 500 kDa	Photothermal heating	ND

A, ion fluxes; B, cytoskeletal remodeling; C, morphological changes; D, DNA damage; E, ER stress; F, delay in cell cycle progression; ND, Not Determined

### Pore forming toxins

Many pathogenic bacteria produce cytotoxic proteins that can perforate the membranes of host cells as part of their virulence [9]. The PM is the main target of most pore forming toxins (PFTs), but some PFTs can also perforate intracellular organelle membranes [10]. A distinction is made between α-PFTs and β-PFTs, depending on whether their secondary structure consists of α-helices or β-barrels, respectively. In all cases, PFTs recognize the target cell by binding to specific ligands, which can be proteins, lipids or sugars. Oligomerization of most α-PFT monomers occurs concomitantly with their insertion into the plasma membrane, while most β-PFTs first form a pre-pore that undergoes conformational rearrangements at cell surface receptors, subsequently leading to their insertion in the membrane [11]. A main group of PFTs that has been exploited for intracellular delivery are the cholesterol-dependent cytolysins produced by Gram-positive bacteria. This family of PFTs allows the formation of pores with a diameter of 25 to 30 nm [12]. Streptolysin O has been used most in vitro [13–15] and allows the delivery of molecules up to 150 kDa [15].

### Direct membrane penetration

PM disruption through direct penetration involves the use of a conduit or vehicle to stab through the membrane. The oldest and best-known example is microinjection, in which the cell membrane is punctured with a miniaturized pipette through which a defined volume of fluid containing the cargo can be injected into the cell. The technique allows delivery of large cargo up until several MDa such as plasmid DNA [16–18]. To increase precision, nanoneedles (or nanowires) have been developed with shorter lengths (< 1 µm) and thinner tips (< 100 nm). This allows injecting compounds even in small subcellular compartments [19]. Arrays of vertically aligned nanowires have been fabricated as well, allowing thousands of cells to be permeabilized simultaneously. Although some reports claim that cells become spontaneously permeabilized by culturing them onto such nanowire arrays [20, 21], others have shown that they enhance endocytic uptake [22, 23]. Still others find that permeabilization only happens when cells are forcefully pushed onto the needles, e.g., by centrifugation [24, 25]. Regardless of the precise mechanism, intracellular delivery is achieved by adding cargo to the medium or by coating the cargo onto nanowire tips [26, 27]. Alternatively, cargo can be pumped through nanostraws, hollow versions of nanowires, thus enhancing delivery efficiency [28, 29].

### Electrical membrane permeabilization

Electrical membrane permeabilization, or electroporation in short, is an approach in which high-intensity and low frequency electrical pulses are used to temporarily destabilize the PM [30, 31]. Transient disruptions in the PM allow the entry of extraneous molecules into the cytoplasm. While smaller molecules can enter by simple diffusion, the entry of macromolecules with a high charge density such as nucleic acids is facilitated by active electrophoresis as well. The dimension and distribution of the created pores typically depend on the magnitude and duration of the applied electric fields [32]. For reversible electroporation, several parameters such as field strength, pulse duration and number of pulses need to be carefully selected to preserve cell viability. If the pulse duration and applied field strength prevent the cell from restoring its integrity, disturbance of the electrolyte balance may trigger cell death [33, 34]. Each cell type demands an optimized electroporation protocol [34, 35], but once determined, up to millions of cells can be treated simultaneously. However, the strong electric fields used in current electroporation techniques may still lead to significant damage or death to cells [36, 37].

### Sonoporation

Sonoporation induces the formation of transient PM pores through acoustic pressure waves, mostly in the ultrasound frequency range (20 kHz to GHz) [38]. The two main mechanisms by which sonoporation perturbs the PM are assumed to be stable cavitation and inertial cavitation (bubble implosion), but other mechanisms have been reported as well [39, 40]. While stable cavitation induces PM disruption through local oscillatory shear forces generated by microstreaming [41, 42], inertial cavitation punctures the membrane via more extreme phenomena, such as potent fluid shear forces generated by microjetting [43, 44]. The addition of gas body ultrasound contrast agents has been shown to drastically improve transfection efficiency compared to ultrasound alone, as they act as cavitation nuclei [45]. A major issue is the random and uncontrolled nature of cavitation events that lead to relatively high levels of cell damage and death [46]. Nevertheless, sonoporation has been shown to hold potential for in vivo applications, where barriers for delivery of therapeutic cargo are situated at the tissue level rather than the cellular level [47].

### Microfluidic cell squeezing

Any shear force induced by severe mechanical deformation may also transiently permeabilize the PM. An elegant implementation drives cell suspensions through a narrow constriction in a microfluidic channel, which causes their rapid mechanical deformation [48]. The constriction volume is tuned to the size of the cells to achieve transient disruption of the PM, which in turn enables the intracellular passage of macromolecules present in the surrounding buffer [49–51]. The PM recovers in a matter of minutes, after which the macromolecules remain trapped in the cytoplasm. While it can operate with very high throughput (~ 1 million cells per second) and has been shown to minimally perturb cell viability and functionality [52], the method is obviously limited to cells in suspension. A practical difficulty is that cells clog at the constriction sites [53]. Moreover, as different cell types differ in size, each cell type requires the development of a new microfluidic device with an optimized constriction volume [54].

### Direct laser-induced photoporation

In direct laser-induced photoporation, also termed *optoporation*, permeabilization is achieved via the direct interaction of a focused laser beam with the PM. Typically, high-intensity femtosecond (fs) laser pulses are employed [55–57]. Membrane pores can be created through photothermal, photomechanical, and photochemical processes and the contribution of each of these processes to membrane poration depends on the laser wavelength, intensity and pulse duration [60–62]. Entry of cargo molecules through the pores occurs by simple diffusion. Even plasmid DNA has been delivered this way with very high transfection efficiency and cell viability [58]. Notwithstanding its effectiveness for single-cell transfections, the general utility has been questioned due to the labor-intensive procedure, high cost and low throughput [58, 59]. On the other hand, via careful 3D-focusing of the focal volume onto the structure that needs to be permeabilized, even intracellular structures such as the nuclear envelope can be specifically perturbed, while leaving the PM intact [60].

### Nanoparticle-mediated photoporation

To enhance throughput and efficiency of direct laser-induced photoporation, the process has been combined with photothermal nanoparticles (NPs). Gold NPs (AuNPs) are used most frequently [2, 3, 61], but other sensitizing particles such as graphene quantum dots [62] and iron oxide-based NPs [5] are bonafide alternatives. Transient permeabilization of the PM is generally achieved by incubating cells with the NPs. After washing away non-associated NPs, the cargo of interest is added and cells are irradiated with a laser, hereby forming pores in the PM at the places where the NPs are present. By using photothermal NPs, the laser energy density needed to create pores is vastly reduced as compared to direct laser-induced photoporation. Consequently, rather than a focused laser beam (as for optoproation), a wide laser beam can be used, which enhances throughput from a few cells per minute to 100.000 cells per second [63]. PM permeabilization is achieved by a variety of photothermal mechanisms that depend on the laser energy and fluence. Upon absorption of continuous wavelength laser light or low intensity laser pulses, the temperature of the NPs increases by tens to several hundred degrees Celsius. The generated heat diffuses into the surrounding environment and causes permeabilization of the nearby PM by a local phase transition of the lipid bilayer or thermal denaturation of integral glycoproteins [64]. Both phenomena result in the formation of short-lived pores with sizes ranging from tens to hundreds of nanometers, depending on the NP size and laser intensity. When the NPs are irradiated with sufficiently high laser fluences, usually from high energy pico- or nanosecond pulsed lasers, the temperature of the NPs can exceed the surrounding liquid’s critical temperature. This results in the vaporization of the liquid around the NPs, leading to the formation of vapor nanobubbles (VNBs). A VNB expands until the thermal energy from the heated NP is consumed [65], after which it collapses and induces mechanical shock waves that generate transient pores in the adjacent membrane. VNB photoporation has been reported to allow intracellular delivery of various compounds, ranging from fluorescent markers [2, 4, 66], over RNA-based macromolecules [2, 3, 67, 68] and plasmid DNA [69, 70] to proteins [71, 72]. Moreover, it can do so in both adherent and suspension cells with high efficiency and low cytotoxicity [3, 66, 69, 73] and it has the precision to target organelle membranes such as the nuclear envelope [74]. Instead of adding photothermal NPs to cells, it is also possible to integrate photothermal features into substrates onto which cells can be cultured [75–77]. This has the advantage that the nanosensitizers will not be ingested by the cell, thereby eliminating a potential safety issue for clinical translation.

## Time-dependent responses to plasma membrane injury in a nutshell

**Fig. 2:**
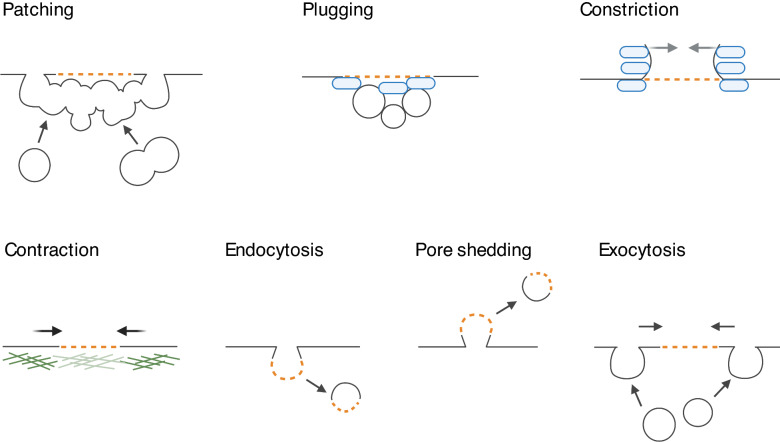
Several mutually non-exclusive mechanisms contribute to plasma membrane repair. Orange dotted lines indicate a breach in the plasma membrane. Annexins are represented in blue and the actin cortex in green

By physically separating the cellular interior from the extracellular environment, the PM creates a biochemically unique environment. Dedicated pumps and transporters tightly control molecular traffic across the PM ensuring a meticulously titrated content. Hence, even the smallest disruptions of the PM barrier can affect cell homeostasis and cell viability. Nanometer-sized ruptures in the PM reseal spontaneously in vitro [78, 79], while closure of larger holes is hampered by membrane tension arising from a combination of intracellular osmotic pressure and cytoskeletal tension (reviewed in [80]). For their sealing, cells rely on active repair machinery (Fig. 2). The instant response to PM disruption is an influx of Ca^2+^, which induces rapid depolymerization of the underlying cortical actin cytoskeleton. The subsequent repair involves the activation of several Ca^2+^-responsive proteins (extensively reviewed in [81]). Irrespective of the size of the PM breach, initial resealing occurs within 30 s [82, 83]. Proteins of the annexin family form a plug [84, 85] and facilitate closure of small (nm) lesions through membrane constriction [86, 87]. Sealing of larger lesions (up to micrometers) relies on fusion of cytoplasmic vesicles with the PM at the injury site (*patching*) [85, 88–90].

Once the injury site is closed, the resealed PM and the underlying cytoskeleton must be restored to their pre-rupture state. For instance, in oocytes and early embryos PM restoration and cytoskeletal repair are intimately linked via the formation and contraction of an actomyosin ring (AMR) minutes post-injury [91]. In somatic mammalian cells on the other hand, there is no such mechanism, and repolymerization of the cortical actin cytoskeleton starts as soon as the PM integrity is reestablished (between 10 and 40 s after disruption [92]). Membrane remodeling to the pre-rupture state takes 60–240 s post-injury [82, 93], or sometimes longer [94]. This includes active removal of damaged membrane, which in the case of small disruptions occurs either by endocytosis [95–97] and/or by pore shedding [98–100]. Exocytosis of endomembrane vesicles at sites adjacent to the injury site probably serves to facilitate tension relief [101–103] and further aids in membrane repair by bringing the membrane edges closer together. Fusion of endomembranes with the PM can replenish lipids and proteins lost at the injury site [94]. For a more comprehensive overview of the various mechanisms of plasma membrane repair, the reader is referred to excellent reviews on this topic [104, 105].

**Fig. 3:**
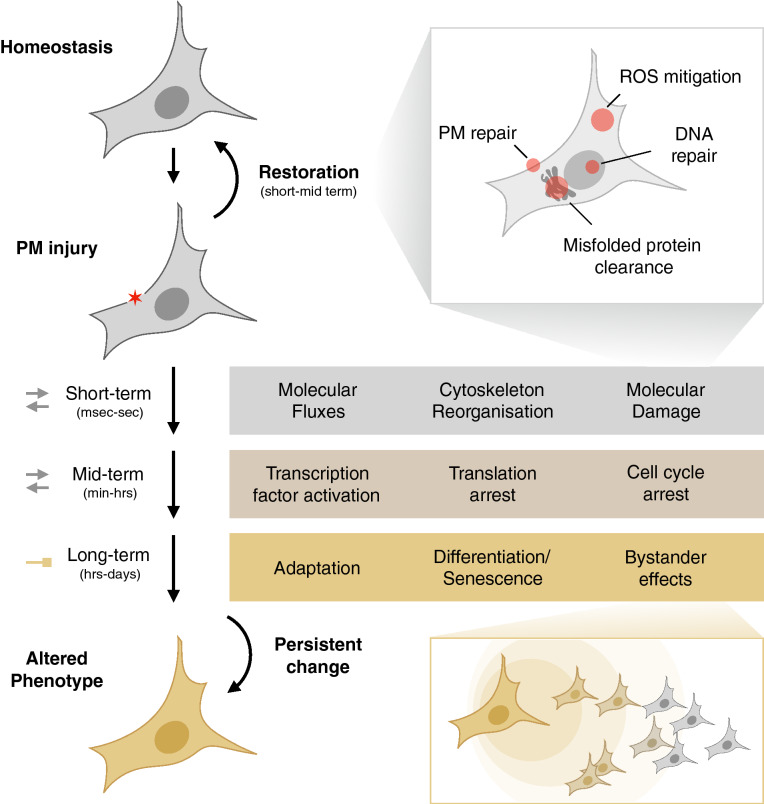
Schematic overview of the different levels and aspects of the cellular response to PM injury. A detailed explanation of the responses can be found in the main text

The consequences of PM disruption do not end with its repair. Cells aim at restoring intracellular homeostasis as well. To do so, they activate several time-dependent processes (Fig. 3). Excessive levels of Ca^2+^ and reactive oxygen species (ROS) [81] need to be eliminated from the cytoplasm, while cell components that were expelled during PM disruption, such as ions and ATP [106, 107], are to be recovered. Similarly, intracellular macromolecules (lipids, DNA or proteins) that are damaged directly (e.g., through heating) or indirectly (e.g., through ROS) during PM injury must be repaired or removed. To prioritize energy consumption for these restorative processes and to avoid transmission of damage to the progeny, cells will trigger an arrest in cell cycle progression [108, 109] and/or translation [110, 111]. If successfully restored, cells will resume their normal functioning, but sustained activation of transcription factors (e.g., CREB) and/or persistent molecular damage may induce lasting changes in gene expression programs and alter cell fate. Some will facilitate adaptation of the cell, to allow it to withstand a subsequent injury, while others evoke phenotypic changes of which the functional relevance is not yet known. In the following sections we will go deeper into the exact molecular rearrangements that take place at the short, mid- to long-term, and give examples of which processes have been documented for nanodelivery techniques.

## A breach in the PM is rapidly repaired but may cause molecular damage and morphological remodeling

### Instant molecular fluxes and their reversal

Disruption of the PM provokes an instantaneous influx of ions, among which Ca^2+^, which is required for efficient repair [112, 113]. When reaching a critical threshold, additional Ca^2+^ may be released from intracellular stores such as the endoplasmic reticulum or the lysosomes [114–116]. Ca^2+^ influx has been documented for various delivery techniques including sonoporation [117, 118], optoporation [116] and nanoparticle-sensitized photoporation [119]. For optoporation, it was shown that the time for reaching the critical threshold level for intracellular Ca^2 +^ release, scaled inversely with the pore size and opening time [116]. For nanoparticle-sensitized photoporation, intracellular stores were found to be the primary source of cytosolic Ca^2+^ increase [119].

Next to Ca^2+^, it has been suggested that oxidants can enter the cytosol upon disruption of the PM [81]. A transient localized increase of ROS at the injury site has been found to facilitate repair [120] by activating specific proteins involved in vesicle fusion [85] and cytoskeletal remodeling [121]. A prolonged general state of oxidative stress however, prevents PM repair, and this can be mitigated by the addition of antioxidants. This was documented for skeletal muscle cells [122, 123], as well as for Chinese hamster ovary cells, which upon electroporation experience a sharp increase in oxidative species [124]. Likewise, increased levels of intracellular ROS were detected after picosecond irradiation of 200 nm spherical AuNPs, which scaled with laser fluence and lasted for min after photoporation [125]. Since ROS are very short lived, such sustained increases in intracellular ROS are more likely triggered by release from endogenous compartments, such as damaged or dysfunctional mitochondria [126].

Several other molecules and ions translocate during PM permeabilization. For instance, a decrease in intracellular K^+^ levels has been reported in cells injured by different PFTs [110, 127]. However, the effects of such fluxes have not yet been investigated in the context of physical membrane disruption techniques.

Once the PM is resealed, the cell will try to restore its intracellular ion levels. Whereas intracellular Ca^2+^ has a vital role in repair of the PM and underlying cytoskeleton, excessive intracellular Ca^2+^ levels inhibit effective PM repair [128] and cause cell death [129, 130]. To avoid this, the cell rapidly lowers intracellular Ca^2+^ levels using cytosolic chelators [131] and ion pumps [132]. The SarcoEndoplasmic Reticulum Calcium ATPase (SERCA) pumps Ca^2+^ back into the lumen of the ER [133], and the mitochondrial uniporter MCU-1 facilitates its uptake by mitochondria [134]. Generally, cytosolic Ca^2+^ levels are restored within minutes after PM disruption [117, 119]. The increase in cytosolic Ca^2+^ In CD4 + T-cells however, cytosolic Ca^2+^ has been found to peak only at 60 min post-nucleofection, before gradually returning to normal levels after 8h [64].

### Cytoskeletal remodeling

Upon PM disruption, the underlying actin cytoskeleton disintegrates rapidly [92, 135], which is thought to be induced by the rapid increase in intracellular Ca^2+^ levels [136]. Ultrasound-microbubble cavitation of the PM has been shown to induce a disruption of the filamentous F-actin network adjacent to the perforation site [137]. Cytoskeletal damage was also reported when treating cells with nanosecond pulsed electric fields [138], but this electroporation regimen is typically used to kill cells such as for the treatment of tumors [139] and thus reflects a condition that is incompatible with cell recovery.

Whereas cortical actin is initially depolymerized upon PM rupture, this is rapidly followed by accumulation of F-actin at the injury site [140]. Repolymerization of F-actin at the injury site is thought to aid in vesicle trafficking [141, 142] and in membrane repair [143–145] and provides structural support to the newly resealed membrane. In sonoporated cells however, the depolymerization of actin fibers was found to further propagate to other parts of the actin cytoskeleton during 60 min and resulted in a granular texture of the actin contents without preferential orientation [137]. One minute after PM pore formation by AuNP-mediated photoporation, a loss in coherency of F-actin fibers was observed, that further decreased 8 min after laser irradiation [146]. This was sometimes found to be accompanied by the formation of membrane blebs. A similar loss in the coherency of F-actin fiber orientation was observed immediately after photoporation of myoblasts grown on a plasmonic pyramid array.

Disassembly of microtubules (MTs) after membrane disruption has also been reported when cells were injured with a glass needle [147] and after sonoporation-induced membrane permeabilization [148]. Within 30 s after membrane disruption however, MTs start to reassemble and approach the injury site by elongation [147]. This is important to allow trafficking of lipids from the trans-Golgi network towards the injury site, which was found to be even more important for membrane resealing when a second insult at the same site was given [147]. The MT network appeared to be fully recovered in most cells within 60 min.

An unexpected recent finding after nanoparticle-sensitized photoporation was a transient upregulation of A-type lamins [149]. Given a concomitant increase in chromatin condensation, this may reflect a global stiffening of the nucleus. Since the loss of A-type lamins sensitized cells to photoporation, the temporary upregulation may aid cells in their recovery from the insult. Interestingly, the upregulation was also observed after cell squeezing and electroporation [52] suggesting it is a generic response to physical PM injury.

### Morphological changes

Upon permeabilization of the PM, changes in the volume of the entire cell and the nucleus have been observed [7, 150]. However, the functional relevance of these changes remains elusive. Swelling or shrinkage may simply reflect a response of the cell trying to counter the osmotic imbalances that were generated through the uncontrolled fluxes of ions and molecules between the cytosol and the extracellular environment during PM injury. Yet, these changes in morphology could trigger mechano-sensitive signal transduction pathways that alter gene expression (see further).

Cell shrinkage was reported to occur within minutes after PM pore formation with several optical permeabilization techniques, including optoporation [150], photoporation with cell-attached plasmonic particles [146] or photoporation with plasmonic substrates [151]. In the case of photoporation of cells with plasmonic substrates, cell shrinkage continued up until 60 min after laser treatment. This decrease in cytoplasm area was accompanied by a decrease in nuclear area, which stabilized after 30 min. Cell swelling was reported for CD4 T-cells subjected to nucleofection with a dramatic expansion in cell size, while the size of the nucleus remained unchanged [7]. Here, it took about one hour for cell morphology to be restored, which corresponds with the time required for effective Ca^2+^ clearance to start [7]. PM poration of myoblasts by optoporation was also shown to induce shrinkage of nuclei and their retraction from the photoporation site [150]. The reorganization of the cytoskeleton upon PM disruption may be directly responsible for changes in nuclear shape and position, as it directly transmits mechanical force to the nucleus via the linker of nucleoskeleton to cytoskeleton (LINC) complex [152]. Shrinkage of both the cell and the nucleus has also been observed in response to sonoporation [108], but this both in cells that were successfully sonoporated and those that were not, suggesting that it may be induced by cavitational forces alone [108].

## Collateral damage may freeze the cell cycle to allow repair

### ROS-induced damage

ROS evoke various stress responses required for cell survival, but—when excessive or persistent—they can also have detrimental effects on cell homeostasis. ROS-induced oxidation of intracellular constituents may cause irreversible damage to all biomolecules. In response to shear stress from bubble oscillation, the level of intracellular H_2_O_2_ markedly increases during 1 h after exposure to ultrasound [118]. Surprisingly, removal of intracellular H_2_O_2_ via the addition of catalase blocked PM permeabilization altogether, suggesting it is a driving factor for successful poration [118]. In fibroblasts cultured on long nanowires, a higher ROS load and DNA damage level were observed, which were attributed to increased respiration rates [153]. Certain combinations of field strength and capacitance of electroporation pulses, also resulted in DNA damage in HL60 cells, although it is not clear whether it is driven by ROS [154].

### ER stress and translational halt

In sonoporated cells, a loss of endoplasmic reticulum (ER) mass was observed over a time period of 6 h post-sonoporation, which may reflect ER dysfunction. Simultaneously, various ER chaperones were upregulated [155, 156]. An upregulation of the ER chaperone HSPA8 was also observed in cortical neural stem cells cultured on nanowire arrays [157], and a set of heat-shock protein encoding genes was found upregulated after nanoparticle-sensitized photoporation [149]. Upregulation of ER chaperones is part of the unfolded protein response (UPR), which is typically induced upon detection of unfolded proteins in the ER and which tries to restore ER homeostasis through various mechanisms. In response to PFTs, activation of the UPR is also observed, but there, it may reflect a response to pathogenic material, rather than an effect of PM disruption [110, 158]. Next to increasing the ER-folding capacity, UPR tries to restore ER homeostasis by limiting the amount of protein that enters. Reduction of global protein synthesis in UPR occurs via the PERK-facilitated phosphorylation of eIF2α [159]. Nucleofection was found to induce this pathway [111], suggesting it triggers ER stress. A transient arrest in protein synthesis was also observed in response to two different PFTs, proaerolysin and listeriolysin, but this was found to be independent of UPR since arrest in protein synthesis was sustained upon silencing of PERK [110].

### Cell cycle stalling

Molecular imbalance or damage may provoke cell cycle arrest. A delay in cell cycle progression is frequently observed in the viable population of various cell types after sonoporation [108, 109]. A concomitant dysregulation was found in the expression of various cyclin and cyclin-dependent kinase checkpoint proteins that are important for cell-cycle progression [109]. The observed accumulation of cells in the G2-M phase was however temporary [109], and cells resume cell cycle progression after some time. Proliferation of T-cells has been shown to be reduced when treated by electroporation for gene editing [3, 160]. One possible reason for this proliferative stall could be DNA damage, but it was not assessed [161]. A delay in cell cycle progression does not seem to occur in response to different types of photoporation. VNB photoporation of Jurkat T cells using nanosecond pulsed irradiation of 60 nm spherical AuNPs showed unaltered proliferation rates up to 5 days post-transfection compared to untreated cells [3]. Primary human T cells and human embryonic stem cells (hESCs) treated with photothermal electrospun nanofibers (PEN photoporation) also did not suffer from a proliferative slowdown [6].

## Even long after restoration of PM injury the downstream effects can linger

The various responses that are induced upon PM injury can trigger long-term alterations in cell fate and phenotype, fuelled by changes in gene expression. These may facilitate adaptation of the cells to the changing extracellular environment, which includes potentiation of injury repair mechanisms to recover faster from a potential second lesion and to increase resistance of the PM to a next insult. However, other less advantageous changes in cell phenotype can be triggered as well. This is a problem for engineered cell-based therapies where the therapeutic effect relies on phenotype-specific functionality.

### Persistent phenotype alterations

Transcription of many genes is regulated directly via Ca^2+^ or indirectly via the Ca^2+^ sensor calmodulin [162–164]. Therefore, if cytosolic Ca^2+^ levels do not return to baseline once the membrane breach has been sealed, they may provoke transcriptional switches. Furthermore, gene expression programs can also be altered by changes in cell volume, as has been observed for different permeabilization techniques (vide supra). Cell proliferation for instance is stimulated by osmotic swelling and is inhibited in osmotically shrunken cells [165]. Regulated cell shrinkage and swelling is also required for cell motility [166] and a disturbed cell volume could therefore complicate cell movement. Cell shrinkage is also a ubiquitous hallmark of apoptosis, independent of the death stimulus [167, 168]. Recently it was found, however, that it is not the cell shrinkage itself, but the ion fluxes that cause cell shrinkage which induce apoptosis [169], implying that cell shrinkage alone does not necessarily lead to programmed cell death. The decrease in cytoplasmic volume is sometimes accompanied by a decrease in nuclear volume as well [170]. These changes in nuclear morphology may simply be the result of osmotic changes in the cells, but they may also be an active response to PM injury. Indeed, changes in nuclear morphology could also activate transcriptional programs to help the cell cope with the consequences of PM injury [171]. As heterochromatin is tethered to the nuclear envelope, changes in nuclear envelope geometry may induce gene expression through alterations in chromatin organization. Changes in gene expression may also be induced through the release of transcription factors that are normally sequestered at the nuclear periphery [172, 173].

Recurrent phenotypic alterations involve activation and adhesion status. In the first 24 h after nucleofection, general transcriptional activity was significantly increased in CD4 + T-cells, indicative of uncontrolled changes in activation status, making these cells no longer useful for studying the functions of naïve T cells [7]. Furthermore, a sustained increase of T cell activation markers CD154 and CD96 has been observed post-nucleofection [7]. Two independent reports also point to the loss of cell adhesion post-nucleofection. Astrocytes showed a reduced re-attachment to the substrate when re-plated after nucleofection [174]. Moreover, the most important differences in the proteome of quail oviduct epithelial cells after nucleofection were related to the absence of cell-adhesion proteins and keratins, indicative of reduced cell adhesion capabilities [8]. Cells cultured on vertical nanowire arrays on the other hand, showed upregulation of CD9, an integral membrane protein that regulates cell adhesion, as well as upregulation of *Rnd2*, which is involved in the organization of the cortical cytoskeleton [157], and *Kifap3*, which is involved in microtubule-based processes [157]. Functional changes related to a loss in polarity have also been observed after nucleofection. The polarity of astrocytes is important for various functions, among which their wound healing response [175]. Upon scrape wounding in vitro, astrocytes polarize and extend protrusions towards the wound. Nucleofected astrocytes displayed a significantly reduced capacity to repopulate the scratched area compared to controls [174]. Finally, nucleofection has been reported to disrupt the fence function of tight junctions in renal epithelial cells, which is responsible for maintaining cell polarity [176]. This resulted in alteration of membrane polarity and mislocalization of transmembrane proteins [176].

Not all techniques however perturb cell homeostasis equally strong as electroporation does. Microfluidic squeezing of human T-cells has been shown to cause minimal aberrant transcriptional responses in contrast to electroporation [52]. Moreover, it was found that T-cells showed undiminished effector responses and homing capabilities after cell squeezing, indicating this technique does not affect T-cell functionality [52]. The impact of nanoparticle sensitized photoporation was found to be modest as well [149], and photoporation with photothermal nanofibers was shown to leave the differentiation potential of hESCs intact, as well as preserving T cell homeostasis and functionality in vitro and in vivo [6].

### Adaptive responses to PM injury

Some of the persistent phenotypic changes may also arm cells for subsequent injury. Progress has been made in elucidating the signaling pathways that are required for adaptation to injury. Whereas PKC appears crucial for resealing an identical PM injury site, PKA activity is required for potentiation against injury at a different site [177–179]. Long-term potentiation of PM repair (24 h) requires cAMP response element (CRE)-mediated gene expression through PKC- and p38 mitogen-activated protein kinase (MAPK)-dependent pathways [180]. The events downstream of CRE-mediated gene expression that facilitate potentiation of injury-induced exocytosis remain to be elucidated. CREB mediates expression of immediate-early gene *c-Fos* [181] becomes upregulated in response to mechanical PM disruption in cell monolayers [182]. Inhibiting PM repair in osteocytes exacerbates *c-Fos* upregulation, while enhancing PM repair rate blunts it [183]. This indicates that at least in osteocytes, *c-Fos* upregulation through PM disruption represents a mechanosensory pathway that allows the bone to adapt to its mechanical environment. Whether *c-Fos* upregulation or that of any other immediate-early gene is required for potentiation of the response to injury, remains to be determined.

A mechano-adaptive response to PM injury has also been observed in circulating tumor cells. More specifically, exposure to fluid shear stress (FSS) induces small disruptions in the PM, which have been found to increase intravascular survival of these cells [184–186]. This increased resistance to membrane permeabilization requires activation of RhoA and actomyosin contractility [187]. What exactly triggers RhoA activation in response to the PM disruption caused by FSS has yet to be determined, but the fact that extracellular Ca^2+^ is essential for FSS resistance in cancer cells [184], suggests that Ca^2+^ influx could activate RhoA directly. It should be noted that membrane disruptions induced by FSS are small (< 12 nm) [187] and it is unclear if the mechano-adaptation holds for larger ruptures as well.

### Long-term bystander effects of injured cells

The response to PM injury is not limited to the injured cell itself. Ca^2+^ can spread to non-injured cells via gap-junctions [188, 189] or via extracellular agonists, such as ATP [190, 191], which trigger secondary release of Ca^2+^ from the lumen of the ER in adjacent cells. Wounding of cell monolayers initiates a Ca^2+^ wave that is propagated outwards from cells at the wound edge, which may prime those cells to proliferate during wound healing. Indeed, propagation of Ca^2+^ to cells further away has been shown to mediate preferential cell growth towards the wound edge [192]. Spreading of Ca^2+^ to adjacent cells may also be important to amplify the production of H_2_O_2_, which serves to attract immune cells to the wound to mount an adequate inflammatory response [193]. A delayed Ca^2+^ wave was for example observed in cells adjacent to sonoporated cells [117, 194]. Ca^2+^ has been found to stimulate endocytosis in adjacent cells as well [43, 195], but the functional role thereof remains to be determined.

Via ATP signaling, neighboring cells are potentiated to induce a membrane repair response, without having the need for a previous insult [196, 197]. This potentiation in other cells than the injured cell is also facilitated on the longer term, via the hydrophobic cell-permeable signaling molecule NO. Long term potentiation in neighboring cells requires CREB phosphorylation through activation of PKG activity [198].

## Conclusion and perspectives

A breach in the PM instantly triggers an influx of Ca^2+^ and disruption of the cytoskeleton, which is often accompanied by changes in cell volume. Resealing of the pores occurs within minutes and the subsequent restoration of the PM both requires Ca^2+^ influx, cytoskeletal reorganization, and in some cases also localized ROS influx. Localized influx of Ca^2+^ and ROS at the injury site can be amplified through additional release from endogenous compartments to trigger additional signaling pathways. Excessive levels of intracellular Ca^2+^ should be removed, since these may disturb various intracellular processes required for normal cell functioning. Levels of intracellular ROS should also be controlled since these can damage various cellular components and perturb multiple pathways. To do so, cells may trigger a transient arrest in cell cycle progression and/or protein translation. Once cellular homeostasis is fully restored, these blocks will be lifted. When the cell does however not succeed to fully recover from the effects of PM injury, a permanent arrest in cell cycle (senescence) and even cell death may be triggered. Depending on the size and the type of PM disruption, the provoked long-term effects will differ, but some interesting similarities have been observed, such as the effect on cell adhesion. Most of the long-term changes of yet unknown functional relevance that are described here have been reported for nucleofection specifically. This is probably because exposing cells to strong electrical pulses can inflict damage to intracellular organelles as well, while the newer PM permeabilization techniques, which often make use of nanotechnology, are designed to limit damage mostly to the PM. As a result, these techniques are likely to have less impact on cell physiology. Nevertheless, further research is needed to understand shared and lasting consequences of PM injury, especially for these more recently developed nanotechnology-based methods. One major aspect is biocompatibility. Some technologies, like photoporation, make use of nanosensitizers that are brought in close contact with cells, and which can stay behind inside cells after treatment. Even though no obvious adverse effects have been reported on the short term, further research is warranted to determine any potential long-term effects of such nanoparticles on cells, especially if they are to be used for therapeutic purposes. Another area for further investigation is cell type-dependency. For, it is plausible that responses to and effects downstream of a membrane breach will differ between cell types based on their size, cycling behavior and molecular markup.

Detailed insight into the consequences of PM injury on cell physiology is especially important for genetic engineering of cells for therapeutic purposes. Indeed, in cell-based therapies and tissue engineering, the genetically modified cells should retain their full capacities in terms of function and proliferation to achieve successful treatment outcomes. Moreover, insight into the cellular response could be exploited to develop strategies to make pore formation easier, or to help the cell cope with the effects of PM injury and avoid unwanted side effects. Simple strategies that are already being used to promote repair consist of supplementing the buffer medium with antioxidants [122, 183] and calcium [15, 199]. The addition of polymers such as polyethyene glycol and poloxamers has been shown to promote membrane resealing as well [200–203], although the exact mechanisms remain to be elucidated. Another approach could consist of boosting the repair machinery directly, e.g., by providing the recombinant counterparts of proteins involved in membrane repair, such as annexins [84, 204, 205]. Next to assisting cells to seal the breach, another strategy to help cells manage PM injury is to prevent changes in cellular volume and composition, using a culture medium of a composition that resembles that of the intracellular fluid [206–208], although this might interfere with the actual repair.

Overall, strategies to help cells cope with PM injury beyond aiding in PM repair are currently limited. Significantly less attention has been given the specific pathways cells employ to cope with longer-lasting downstream effects of PM injury such as ER stress and cell cycle arrest. Exactly these pathways may be considered for combination treatments and for improving cell fitness after PM disruption. Future research could benefit from a more comprehensive analysis of these effects and their modulation, to obtain a holistic view on the cellular response to PM injury and to advance the field of PM disruption-mediated intracellular delivery.

## Data Availability

Not applicable.

## Abbreviations

AuNP: Gold nanoparticle
ER: Endoplasmic reticulum
FSS: Fluid shear stress
PEN: Photothermal electrospun nanofibers
PFT: Pore-forming toxin
PM: Plasma membrane
ROS: Reactive oxygen species
VNB: Vapor nanobubble
